# What Lies Behind Diagnostic Labels? High Intra-Individual Variability Is the True Cognitive Signature of University Students with Specific Learning Disorders

**DOI:** 10.3390/brainsci16040404

**Published:** 2026-04-10

**Authors:** Sara Zonca, Marzia Lucia Bizzaro, Luisa Girelli

**Affiliations:** 1 Department of Brain and Behavioral Sciences, University of Pavia, 27100 Pavia, Italy; sara.zonca01@universitadipavia.it; 2 Department of Psychology, University of Milano-Bicocca, 20126 Milano, Italy; marzia.bizzaro@unimib.it

**Keywords:** specific learning disorder, university students, cognitive profile, transdiagnostic approach, comorbidity, domain-general components, intra-individual variability

## Abstract

**Highlights:**

**What are the main findings?**

- University students with Specific Learning Disorders consistently show higher reasoning abilities compared to cognitive efficiency, with working memory emerging as a persistent core weakness. However, there is marked individual variability that undermines a unitary deficit model.
- Latent Profile Analysis identified two distinct cognitive subgroups (“High” and “Low” profiles) that do not align strictly with traditional diagnostic labels, showing that intra-individual discrepancies exist across all diagnostic categories.

**What are the implications of the main findings?**

- Traditional categorical labels have limited explanatory power in adulthood due to high cognitive heterogeneity, suggesting that a “one size fits all” approach is particularly inadequate when transitioning out of childhood.
- Clinical and educational support should shift from label-based interventions toward dimensional, profile-based models that address the specific cognitive strengths and vulnerabilities of each student.

**Abstract:**

**Background/Objectives**: Specific Learning Disorders are lifelong neurodevelopmental conditions that persist in adulthood, yet research has traditionally focused on children. In adults, there is significant heterogeneity in cognitive profiles and a lack of consensus on how to operationalize these disorders. This study aims to map the variability in cognitive functioning in university students with Specific Learning Disorders and investigate whether cognitive profiles differ across diagnostic categories and comorbidities. **Methods**: A retrospective analysis was conducted on the clinical documentation of 166 university students with a diagnosis of Specific Learning Disorders. Participants were categorized into three subgroups: predominant reading disorder, predominant arithmetic disorder, and mixed learning disorder. Cognitive functioning was assessed using Wechsler scales indices. Data were analyzed using linear mixed-effects models and Latent Profile Analysis. **Results**: Across the sample, reasoning abilities were significantly higher than cognitive efficiency, with working memory consistently emerging as a core weakness. The mixed-disorder group exhibited the lowest cognitive scores and the greatest working memory deficits. Latent Profile Analysis identified two distinct latent subgroups: a “Low Profile” characterized by weaker working memory and a “High Profile” characterized by stronger reasoning and balanced efficiency. Diagnostic labels were only partially aligned with these profiles; while the mixed-disorder group was overrepresented in the “Low Profile,” substantial intra-individual variability existed across all diagnostic categories. **Conclusions**: The findings suggest that traditional categorical labels for Specific Learning Disorders have limited explanatory power in adulthood, given the high heterogeneity of cognitive functioning. Cognitive weaknesses, particularly in working memory, persist even in high-achieving university students. Clinical and educational support should shift from a label-based approach toward a dimensional, profile-based model to better address the unique strengths and vulnerabilities of adults with Specific Learning Disorders.

## 1. Introduction

Specific Learning Disorders (SLDs) are neurodevelopmental conditions characterized by persistent and selective deficits in academic skills, i.e., reading, writing, and calculation, despite adequate intellectual functioning and access to appropriate learning opportunities. According to DSM-5, the international prevalence of SLDs in children ranges from 5% to 15%. While prevalence rates among adults remain less clearly defined, current estimates suggest a figure of approximately 4%. Furthermore, SLDs exhibit a higher incidence in males compared to females, with reported sex ratios ranging between 2:1 and 3:1 [1].

From a sex and gender perspective, the existing literature reveals distinct dimorphic patterns in cognitive and behavioral manifestations. While female children often demonstrate a literacy advantage in spelling and reading fluency, findings in mathematics are more heterogeneous, with some evidence suggesting a higher prevalence of challenges among females [2,3,4]. In contrast, the observed lower reading performance in males is frequently attributed to their overrepresentation at the lower end of test score distributions, a gap mediated by sex-specific differences in processing speed, inhibition, and verbal reasoning [2]. Additional findings on dyslexia also suggest better verbal working memory and conceptualization, and orthographic and visuospatial coding in females [5].

By definition, as with any other neurodevelopmental condition, SLDs are lifelong conditions: typically, difficulties manifest in the early years of formal education and persist across development and the school years into adulthood, affecting educational and occupational outcomes across the lifespan [6].

SLDs represent a heterogeneous group of cognitive disorders with many subtypes and no adult-specific definition [7], as reflected by highly variable outcomes: adults with SLDs range from highly successful professionals to marginally adjusted individuals, suggesting a wide range of severity [6,8].

Moreover, comorbidity is common, further increasing variability in functional and adaptive profiles. Among the most common conditions associated with SLD are Attention-Deficit/Hyperactivity Disorder (ADHD) and language disorders, as well as psychopathological conditions (e.g., anxiety, depression, personality disorders), whose expression worsens over time [9,10]. In terms of intellectual profile, variability is even more pronounced, since intellectual disability is the only exclusion criterion within DSM-5 [1], allowing for SLD diagnosis in borderline profiles (i.e., Full-Scale IQ 70 < x < 85) as well as in intellectually gifted individuals. Indeed, twice-exceptionality is a well-recognized and relatively frequent condition characterized by the co-occurrence of neurodevelopmental disorders, among which are SLDs, with higher cognitive functioning [11,12]. Thus, when referring to adults with SLD, “one size does not fit all” [7] appears to be the most cautious approach to avoid oversimplifications and incorrect generalizations about long-term, extremely diverse outcomes.

A further challenge concerns SLD recognition in adulthood, given that, despite the issue being raised in the literature [7,13], there is no agreement on how to operationalize SLD in adults [14]. In particular, the variety and complexity of extracurricular contexts multiply daily demands and challenges, making it difficult to determine when and to what extent SLD may contribute to additional fatigue. In fact, research on SLD has so far mainly focused on children, since expression of the disorder typically manifests over the school years. It follows that efforts have long centered on early identification and remediation, with little attention to the long-term stability of learning deficits and profile modifications towards adulthood [15].

In recent years, the issue of SLD in adults has attracted increasing attention across educational, clinical, and scientific contexts. There is growing evidence that SLDs are associated with enduring cognitive differences that persist into adulthood [16,17]. Thus, it is urgent to understand the reasons for the divergent developmental trajectories among individuals with SLD. Not surprisingly, variability in cognitive resources may well account for differences in the attainment of age-appropriate learning skills and in functional adaptation to adult life challenges. The predictive value of cognitive functioning in characterizing the learning profiles of SLD children is well established [18]; the pattern of cognitive strengths and weaknesses may help distinguish a transient difficulty from a true disorder [3,19]. There is no reason to believe that this would not extend to adulthood.

### 1.1. Adults with SLD: The University Population

The adult population with SLD is inherently more difficult to identify and monitor over time than the scholastic-age population. In fact, during school years, learning difficulties become clear within a highly structured and regulated system where academic requirements are standardized and performance is continuously monitored. This context encourages schools and families to refer children to clinical and diagnostic services, making the identification of children with SLD relatively systematic. In adulthood, motivational and psychological factors may play a critical role in masking difficulties, in developing adaptive compensatory strategies, or in avoiding challenging activities [20,21].

Moreover, beyond the increasing promotion of inclusive policies, higher-educational pathways are the only contexts that explicitly accommodate young adults with SLD [22,23]. Thus, individuals who, regardless of underlying reasons, skipped early diagnosis are unlikely to be recognized as atypical learners in their post-secondary pathways.

For this reason, most research on SLDs in adulthood focuses on university populations. University is one of the few settings in which adults with SLDs are required to produce formal diagnostic documentation, typically to access accommodation and compensatory measures [24]. This makes university students with SLDs more easily identifiable and recruitable than other adults, although they constitute a highly selected subgroup of the population with SLDs, i.e., those who have successfully accessed higher education, which inherently biases samples toward individuals with relative motivational and academic strengths.

Yet, even within university students with dyslexia, targeted investigations have shown that cognitive heterogeneity is high, with some of them even outperforming typical readers in nonverbal measures [25] and with most of them showing partial remediation or adoption of compensatory strategies [25,26]. In particular, research on developmental dyslexia suggests that compensation may index a discrepancy between the perceived ability of an individual (i.e., degree of observable behavioral symptoms) and actual ability, as exhibited in underlying cognitive and/or neural function [27,28]. Accordingly, university students with SLD may constitute a rather specific group of individuals with a history of underachievement at school [29], but they offer an important window into the adult expression of learning difficulties in a highly educated context [30].

In Italy, government reports indicate that, in recent years, there has been an increase in SLD cases in middle and high schools [31], partly expected given the introduction of normative regulation from 2010. Italian Law 170/2010 formally acknowledges dyslexia, dysorthographia, dysgraphia, and dyscalculia as clinical conditions allowing access to compensatory measures and accommodations in educational settings, including universities (the Italian Ministry of Education reports ~6% of school-aged learners with SLD) [31]. This legislative framework has contributed to increased attention to SLD within the education system, although higher-educational contexts remain relatively unexplored.

While decades of research have deepened understanding of SLDs in childhood, the lifelong functioning of SLD individuals has only recently been the object of targeted studies. Large-scale studies on cognitive profiles in school-aged SLD individuals typically do not examine age-related differences, suggesting consistency across school years in cognitive strengths and difficulties patterns associated with this clinical condition [32]. Similarly, evidence from large-scale cognitive assessments suggests that adult manifestations of SLD share core characteristics with childhood profiles but also show patterns specific to maturational and experiential factors in adulthood [16,33,34].

### 1.2. Intellectual Profiles of Adults with SLD

Due to incremental attention to the lifelong impact of SLD, the recent literature focuses on the cognitive functioning in adults with SLD using standardized measures such as the Wechsler Adult Intelligence Scale, Fourth Edition (WAIS-IV). Pizzigallo [16] investigated WAIS-IV profiles in adults with SLD and found higher reasoning-related scores (General Ability Index, GAI) alongside lower working memory (WMI) and processing speed (PSI) scores. This fragmented profile mirrors patterns observed in childhood and shows persistent cognitive weaknesses in adulthood. Overall, this study strengthens the discriminatory power of the WAIS-IV profile in distinguishing adults with SLDs from those without [16].

Converging evidence from studies on university students with reading-related SLD, i.e., dyslexia, indicates enduring difficulties in WMI and PSI relative to peers, with nonverbal reasoning (Perceptual Reasoning Index, PRI) and verbal comprehension (Verbal Comprehension Index, VCI) often relatively preserved [34,35,36,37]. These patterns hold even among highly educated adults, suggesting that cognitive weaknesses associated with SLD may not be fully compensated by academic exposure alone [25].

Research specifically addressing dyslexia and spelling disorders further confirms the persistence of domain-specific challenges. For example, some studies report lasting reading and writing difficulties in adults with dyslexia despite high levels of education and academic engagement [34,35,36,37]. These findings emphasize that SLD-related cognitive difficulties extend beyond childhood and school settings into adult life.

In contrast, relatively little is known about dyscalculia in adults [38]. This gap may reflect heterogeneity in the clinical assessment and operationalization of mathematical disorders in adulthood as well as the long-standing emphasis on reading over other learning impairments in research.

### 1.3. Complexity and Comorbidity in SLD

The dimensional approach to neurodevelopmental disorders introduced by DSM-5 [1] warns against oversimplifying the clinical heterogeneity of SLD. This term is recognized as an umbrella label that requires further specification in terms of the interested domains of learning (reading, spelling, writing, or calculation) and degree of severity. Most interestingly, evidence from developmental samples indicates that mixed profiles and comorbidities are extremely common: in Italy, approximately 50% of SLD diagnoses consist of mixed disorders, with selective deficits being the exception rather than the rule [32,39,40]. This complexity underscores the limited informative value of treating SLD as a unitary category, as also highlighted by Pizzigallo et al. [16], in adult SLD research [41].

Despite this complexity, the clinical and theoretical value of identifying distinct cognitive subtypes within adults with SLD has been acknowledged for several decades. In one of the earliest systematic investigations of learning disorders in adulthood, McCue et al. [42] used standardized psychometric measures to delineate heterogeneous cognitive profile patterns among adults referred for learning difficulties, demonstrating that adult SLDs cannot be adequately captured by a unitary deficit model. This seminal work provided early evidence that meaningful interindividual variability exists across core cognitive domains, supporting the notion that subtype-oriented approaches are essential for understanding the adult expression of SLD and for linking diagnostic labels to underlying cognitive functioning.

### 1.4. The Present Study

Building on this literature, the present study aims to map the variability in cognitive functioning in university students with SLD and to investigate differences in cognitive profiles across diagnostic categories and comorbidities.

Following previous studies [22,29,43], we reach our purpose by reviewing the documentation provided by SLD students to the university office during enrollment. Once enrolled, students can refer to the university’s SLD and disability services to obtain academic accommodations based on the diagnostic profile derived from the documentation. Retrospective clinical documentation is a valuable resource but is inherently imperfect. Inconsistency and variability across professionals and institutions make retrospective documental analysis a challenging process. In fact, despite national guidelines for clinical practice specific to SLD [44], clinical services greatly differ in both assessment procedures (e.g., adopted protocols, clinical tools, reference to cut-off scores or performance range) and reporting (e.g., details and structure of the clinical documentation). This challenge is faced overseas, where previous work has highlighted a disconnection between clinical assessment reports and university documentation requirements, reflecting long-standing challenges in linking diagnostic data with functional academic outcomes [29].

Bearing these limitations in mind, we leverage available clinical data to improve our understanding of cognitive heterogeneity in the adult SLD population. In particular, using retrospective clinical documentation, this research examines cognitive functioning as reflected by the Wechsler indices scores associated with the learning disorder profiles. In fact, though the cognitive profile of the adult SLD population has been targeted in previous studies [16,20,33,41], to our knowledge, little, if any, effort has been so far directed to consider learning profiles, rather than a unique SDL status, as an independent variable. Operationally, the learning profile may be detected by referring to nosographic diagnoses, as indicated by the ICD-11 coding system [45], bearing in mind that, in the vast majority of cases, multiple codes are assigned to the same individual. Although this is better than considering adults with SLD as a whole, this categorization is, in this age group, partly simplistic. In fact, while at an early stage of schooling, describing an SLD in terms of instrumental skills resistant to automatization (i.e., reading, spelling, and calculation) may be a good starting point for developing intervention strategies, such labelling is, in young adults, of limited clinical utility. Over time, any persistent difficulties in reading, writing, and/or arithmetic change expression, making clinical judgement (i.e., anamnestic data, adaptive functioning, perceived difficulties in daily life, etc.) even more informative for the diagnostic process [20]. Yet, due to the retrospective nature of our study, we refer to the ICD-10 coding system [46], which is required by law in every diagnostic report for SLD to obtain clinical information on our participants. Given the lower frequency and lower impact of isolated spelling disorders and dysgraphia in young adults, we clustered individuals with SLD into three subgroups: predominant reading disorder, predominant arithmetic disorder, and mixed disorder. We explored cognitive profiles based on SLD diagnostic subgroups and derived latent subgroups based on these profiles. Specifically, cognitive profiles were examined within and between different SLD subgroups, exploring discrepancies in Wechsler optional (i.e., GAI–CPI, Cognitive Proficiency Index) and core (i.e., VCI–PRI, WMI–PSI) indices. Then we exploited latent analysis to identify latent cognitive profiles and evaluate their potential alignment with diagnostic classifications.

In sum, our study sought to explore: (1) differences in cognitive profiles across SLD subgroups; (2) intra-individual variability between pairs of indices (i.e., VCI–PRI and WMI–PSI); (3) frequency of index discrepancies (≥15 scores) across SLD subgroups; and (4) the presence of latent subgroups considering individual cognitive profiles.

Based on the previous literature, our hypotheses were first to detect an overall discrepancy between the GAI and CPI indexes, as reported in SDL children across different learning profiles [32] and confirmed in dyslexic adults [34] and unspecified SLD adults [16]; following what is observed in SLD children, we expected this discrepancy to be reduced in the mixed-disorder group due to the overall lower GAI of this latter group compared to the other two groups [32]. As for the core indexes, we did not expect overall significant discrepancies within VCI and PRI indexes, apart from the AD group, where a difference may be expected in favor of VCI. This is followed by the assumption that the overall overlapping average profile groups in SLD children and adults [16] extend to specific learning profiles, i.e., a specific disorder of arithmetical skills [32]. Additionally, we expect WMI to be the lowest index for all groups.

With regards to cognitive patterns within diagnostic groups, we expect a high degree of intra-individual variability within each diagnostic group, challenging the notion of a single, deterministic cognitive profile for SLD. We argue that the relatively modest numerical differences often observed between pairs of indices at the aggregate level do not necessarily reflect a uniform or “typical” cognitive constraint shared by all individuals within a diagnostic category. Instead, these stable averages are likely the mathematical result of highly divergent individual profiles. In fact, while the literature consistently reports stable group-level trends in the cognitive profiles of adults with SLD (e.g., PSI > WMI and VCI > PRI) [16,32], these studies typically focus on mean scores, which may paradoxically mask profound individual heterogeneity. To date, research has seldom addressed within-group cross-tabulations of index directions, such as the specific proportion of individuals. By explicitly investigating variability at an individual level, this study aims to demonstrate that the apparent stability of the SLD cognitive “signature” is secondary to a much more complex and idiosyncratic internal variability.

Finally, on this ground, we expect that cognitive subgroups identified by the latent profile analysis do not strictly correspond to learning profile groups, suggesting low informative value of diagnostic labels. Traditional nosographic labels are primarily descriptive of behavioral symptoms rather than the underlying cognitive mechanisms. While clinical categories such as dyslexia or dyscalculia focus on domain-specific deficits, neurodevelopmental research suggests a significant overlap in domain-general vulnerabilities, such as executive functions and processing efficiency [47]. Therefore, we expect that the subgroups emerging from the Latent Profile Analysis (LPA) will reflect functional phenotypes characterized by specific combinations of strengths and weaknesses that transcend diagnostic boundaries. This would imply shifting the clinical focus from a categorical approach that carries low informative value regarding the actual cognitive resources of an individual toward a dimensional, profile-based model, which is more aligned with the personalized support required in higher education.

## 2. Materials and Methods

### 2.1. Participants

A total of 166 university students (107 females and 59 males; mean age at last assessment = 17.36 years, SD = 2.04) participated in the study. All students had been previously diagnosed with SLD by an authorized clinical service using the diagnostic criteria as outlined in the ICD-10 [46] and DSM-5 [1] and following the standard psychodiagnostics assessment guidelines [44]. All participants were first-year students at the University of Milano-Bicocca. Given that this is a large public university located in the metropolitan area of Milan, the sample can be considered relatively homogeneous in terms of socio-economic status. Regarding the academic distribution of the sample, 30% of participants were recruited from the School of Sciences (comprising six departments) followed by the Department of Educational Sciences (16%). The cohort further included students from the School of Economics and Statistics (comprising three departments) (14%), the Department of Law (12%), and the Department of Medicine and Surgery (11%). Smaller subsets of the population were represented by the Department of Sociology (10%) and the Department of Psychology (5%). As the research involved a secondary analysis of archival data originally collected for assessment purpose by authorized clinical centers, ethical approval from an Institutional Review Board was not required. The data were fully anonymized for the research.

### 2.2. Procedure

Students were identified through the following inclusion criteria: (a) 18 years of age or older; (b) students who attended Italian high school; (c) absence of disabilities or other neurodevelopmental disorders; (d) enrolled to a three-year or to a five-year degree at the University of Milan-Bicocca; (e) previously diagnosed with SLD; and (f) provided clinical documentation during enrollment.

This search was conducted in April 2025 and provided 368 eligible participants who underwent a further selection based on the following exclusion criteria: (a) documentation dated before the thirteenth year of age (transition year to high school) (*n* = 14); (b) absence of cognitive assessment (*n* = 56); (c) cognitive assessment by means of older versions of Wechsler scales (i.e., third version or below) or of other instruments (e.g., Raven matrices) (*n* = 81); and (d) borderline intellectual functioning (BIF) as reflected by a GAI blow 85 (*n* = 13) [48,49]. We identified 166 students who met the study’s criteria. We culled descriptive and norm-referenced data from each student’s documentation, including: (a) gender; (b) age; (c) most recent SLD diagnosis; (d) grade of most recent SLD diagnosis; (e) SLD diagnosis and class of first diagnosis (when available); and (f) norm-referenced scores for the fourth version of the Wechsler scale.

Most participants reported norm-referenced scores using the Italian version of the Wechsler Adult Intelligence Scale, Fourth Edition (WAIS-IV) [50] (*n* = 123); one-third reported scores from the Wechsler Intelligence Scale for Children, Fourth Edition (WISC-IV) [51] (*n* = 42). Following the Italian regulation, participants’ diagnoses were associated with one or more learning disorders, adopting the ICD-10 coding system [46] as follows: specific reading disorder (F81.0), specific spelling disorder (F81.1), specific disorder of arithmetical skills (F81.2), and other developmental disorders of scholastic skills (dysgraphia) (F81.8). To our purpose, participants were divided into three subgroups: (a) predominant reading disorder (RD), including F81.0 diagnosis with or without F81.1 and/or F81.8 comorbidity (*n* = 79); (b) predominant arithmetic disorder (AD), including F81.2 diagnosis with or without F81.1 and/or F81.8 comorbidity (*n* = 24); and (c) mixed leaning disorder (MD), including F81.0 and F81.2 diagnosis with or without F81.1 and/or F81.8 comorbidity (*n* = 63). Subgroups were age-matched (*p* = 0.322) but not gender-matched (*p* < 0.001), as shown in Table 1.

### 2.3. Instrument

The Italian adaptations of WAIS-IV and WISC-IV, including the four main indexes (VCI, PRI, WMI, and PSI) and the two additional indexes (GAI and CPI), were used. VCI (based on Similarities, Vocabulary, and Comprehension subtests) and PRI (based on Block Design, Picture Concepts, and Matrix Reasoning subtests for WAIS-IV and based on Block Design, Matrix Reasoning, and Puzzle subtests for WISC-IV) were combined in GAI. WMI (based on Digit Span, Letter–Number Sequencing, and Arithmetic subtests) and PSI (based on Coding and Symbol Search subtests) were combined to form CPI. To enhance profile interpretation, WAIS-IV and WISC-IV formalized the use of GAI and introduced CPI, allowing clinicians to differentiate reasoning abilities from cognitive efficiency processes [52]. Following the recent literature showing that Full-Scale IQ (FSIQ) may be less informative or even misleading, especially when WMI and PSI are significantly lower than VCI and PRI, as in the case of SLD, we did not report this index [32,53,54,55]. See Table 1 for descriptives of indices in the overall sample and in the diagnostic groups.

### 2.4. Analysis

All statistical analyses were performed using R (version 2025.09.1+401) for Windows. To analyze pairwise differences between cognitive indices across diagnostic subgroups, a linear mixed-effects model (LMM) was employed, with index scores as the dependent variables. The model specified effects for group (AD vs. RD vs. MD), index (GAI vs. CPI, VCI vs. PRI, WMI vs. PSI), and their interaction, including a by-participant random intercept to account for repeated measures across indexes. Bonferroni-adjusted post hoc comparisons were conducted for significant effects. Partial eta-squared (η^2^p) values were computed to quantify the effect size of the differences between indexes and groups. Before fitting the models, all dependent variables were examined for normality and homogeneity of variances. The Shapiro–Wilk test was used to assess the normality of score distributions, and homogeneity of variance was evaluated accordingly. All assumptions were met for each variable (all *p* > 0.05).

In the absence of a control group, one-sample *t*-tests were conducted to compare participants’ WAIS-IV index scores (GAI, CPI, VCI, PRI, WMI, PSI) against the normative mean of 100. For each index, the null hypothesis tested whether the sample mean significantly differed from the normative value. Two-tailed tests were applied. For each test, 95% confidence intervals (CIs) were computed. Statistical significance was additionally evaluated by examining whether the normative value (100) fell within the CI: if the interval did not include 100, the mean was considered significantly different from the normative population. Effect sizes were calculated using Cohen’s *d*. To control for multiple comparisons across the six indices, Bonferroni’s correction was applied to *p*-values. This approach provides an indirect estimate of deviation from normative performance; however, it does not substitute for comparisons with an actual control group, as it does not account for variability in the normative population.

Further analysis focused on WAIS-IV cognitive assessments (*n* = 123), i.e., the most recently assessed subsample of participants (see Table 2 for descriptives of the subsample). We investigated intra-individual variability and explored differences across pairs of main indices (VCI–PRI and WMI–PSI) by calculating subject-level difference scores to quantify both the direction and magnitude of the difference. For each difference score, we tested whether the subsample mean difference deviated significantly from zero using two-sided one-sample *t*-tests. We then calculated the proportion of those showing clinically significant discrepancy (i.e., ≥15 standardized points difference between indices) within each diagnostic group and performed a chi-squared test of independence to explore whether the presence of clinically meaningful intra-individual discrepancies significantly differed across diagnostic groups.

We sought to classify reports into homogeneous subgroups to investigate whether unobserved subgroups of cognitive functioning could emerge from WAIS-IV index scores. To do so, we conducted Latent Profile Analysis (LPA) using the tidyLPA package (version 2.0.1) in R [56]. LPA analysis classifies individuals into their most probable “profile”. We estimated models ranging from 1 to 8 profiles. Model selection was based on information criteria (AIC, BIC, SABIC), supplementary indexes (AWE, CLC, KIC), the analytic hierarchy process suggested by Akogul and Erisoglu [57], and inspection of minimum class size. Following the recommendations outlined by Watkins and Canivez [58], the latent profile models were estimated assuming local independence and homogeneous within-profile variances. These constraints are commonly adopted to reduce model complexity and ensure robust estimation.

Once the optimal profile solution was identified, a series of post hoc analyses were performed to characterize the emerging profiles. Between-profile comparisons analyses were performed to compare the two profiles on WAIS-IV indices’ scores (VCI, PRI, WMI, PSI). The appropriate inferential statistics and effect size measures were selected based on assumption verification (i.e., normality and homogeneity of variance). Within-group analyses were also performed to examine differences between WAIS-IV indices scores (VCI, PRI, WMI, PSI) within each identified profile. A repeated-measures analytical framework was adopted after examining the fulfillment of parametric assumptions (i.e., normality, sphericity). Where global tests reached significance, post hoc pairwise comparisons were conducted to identify specific index differences. Finally, a chi-squared test was performed to determine the proportion of individuals belonging to each cognitive profile across diagnostic groups.

## 3. Results

### 3.1. Linear Mixed-Effect Model

**Figure 1:**
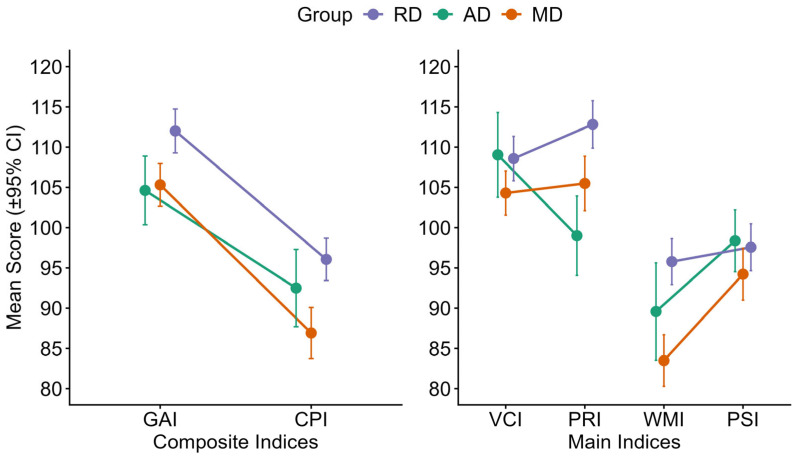
WAIS-IV and WISC-IV indices’ scores for RD, AD and MD groups. *Note*. RD = predominant reading disorder; AD = predominant arithmetic disorder; MD = mixed learning disorder; GAI = General Ability Index; CPI = Cognitive Proficiency Index; VCI = Verbal Comprehension Index; PRI = Perceptual Reasoning Index; WMI = Working Memory Index; PSI = Processing Speed Index. Error bars indicate 95% confidence intervals.

Figure 1 presents group scores for the main and additional WAIS/WISC-IV index scores (GAI, CPI, VCI, PRI, WMI, PSI) across diagnostic groups. The linear mixed-effects model assessing GAI and CPI discrepancies revealed a significant main effect of index (F(1, 163) = 168.25, *p* < 0.001, η^2^p = 0.51), suggesting higher mean scores in GAI than CPI (t(163) = 12.97, *p* < 0.001). There was also a significant main effect of group (F(2, 163) = 11.84, *p* < 0.001, η^2^p = 0.13): overall mean scores were significantly higher in the RD group compared to the MD group (t(163) = 4.78, *p* < 0.001). No significant group × index interaction was found (*p* = 0.14), indicating that the index effect is consistent across groups.

Regarding VCI and PRI score differences, no significant effect of index was found (*p* = 0.24), indicating that, across the entire sample, mean scores on ICV and IRP do not differ significantly. The effect of group was significant (F(2, 163) = 7.03, *p* = 0.001, η^2^p = 0.08): overall, RD mean scores were significantly higher than both AD (t(163) = 2.75, *p* = 0.01) and MD (t(163) = 3.30, *p* = 0.003) mean scores. However, a significant group × index interaction (F(2, 163) = 8.77, *p* < 0.001, η^2^p = 0.10) revealed distinct patterns of scores among groups. Specifically, in the RD and MD groups, scores on VCI were lower than those on PRI. In the AD group, an opposite pattern emerged, with mean VCI scores higher than PRI scores. However, post hoc *t*-tests (Bonferroni-corrected) showed that the within-group differences across the two indexes were significant only in the AD group (t(163) = 3.36, *p* = 0.014). In the RD (*p* = 0.16) and MD (*p* = 1) groups, no significant differences across the two indexes were found, suggesting a relative balance between verbal comprehension and perceptual reasoning. Analysis of performance between groups revealed significantly higher PRI scores in the RD group than in both the AD (t(163) = 4.66, *p* < 0.001) and MD (t(163) = 3.41, *p* = 0.011) groups.

Analyzing WMI and PSI discrepancies, a significant effect of index (F(1, 163) = 24.57, *p* < 0.001, η^2^p = 0.13) was observed, with significantly higher scores in PSI compared to WMI across the overall sample (t(163) = 4.96, *p* < 0.001). The effect of group was also significant (F(2, 163) = 10.44, *p* < 0.001, η^2^p = 0.11), revealing that the RD mean score was significantly higher than the MD mean score (t(163) = 4.56, *p* < 0.001). Additionally, a significant group × index interaction (F(2, 163) = 5.69, *p* = 0.004, η^2^p = 0.07) was found, suggesting that the magnitude of the difference between the two indexes varies as a function of clinical profile. Pairwise comparisons (Bonferroni-corrected) revealed that the interaction effect was driven primarily by the MD group, in which WMI scores were significantly lower than PSI scores (t(163) = 5.25, *p* < 0.001). No significant within-group differences between WMI and IVE emerged for the AD group (*p* = 0.13) or the RD group (*p* = 1).

Between-group comparisons revealed significantly higher WMI scores in the RD group compared to the MD group (t(163) = 5.60, *p* < 0.001).

One-sample *t*-tests were conducted to compare participants’ WAIS-IV index scores against the normative mean of 100: descriptive and inferential analyses revealed systematic deviations from the normative mean across all indices.

One-sample *t*-tests showed relatively comparable or higher verbal and perceptual reasoning abilities with VCI (t(165) = 7.42, *p* < 0.001, *d* = 0.58, 95% CI [105.15, 108.89]), PRI (t(165) = 7.30, *p* < 0.001, *d* = 0.57, 95% CI [105.87, 110.22]), and GAI (t(165) = 9.05, *p* < 0.001, *d* = 0.70, 95% CI [106.57, 110.24]) showing significantly higher scores than the normative mean.

In contrast, WMI (t(165) = −8.77, *p* < 0.001, *d* = −0.68, 95% CI [88.02, 92.42]), PSI (t(165) = −3.62, *p* < 0.001, *d* = −0.28, 95% CI [94.46, 98.37]), and CPI (t(165) = −7.90, *p* < 0.001, *d* = −0.61, 95% CI [90.10, 94.06]) were significantly lower than the normative mean, highlighting marked weaknesses in working memory and processing speed. Consistent with these findings, none of the 95% confidence intervals included the normative value of 100, confirming that all indices significantly deviated from population expectations. These findings should be interpreted with caution, as the comparison relies on a fixed normative value rather than an empirically matched control group, potentially inflating estimates of group differences.

Participants assessed with WAIS-IV (i.e., the most recently assessed subsample of participants) (*n* = 123) were then grouped to examine intra-individual variability between pairs of indices (VCI–PRI and WMI–PSI).

Descriptive analyses revealed substantial variability across participants. For VCI–PRI, difference scores ranged from −34 to +48 points, with a mean difference of −0.15 (SD = 16.17), indicating that discrepancies were widely distributed in both directions and not centered around a systematic group-level bias. For WMI–PSI, differences ranged from −53 to +28 points, with a mean difference of −5.96 (SD = 16.90), suggesting a tendency toward lower WMI relative to PSI.

One-sample *t*-tests were used to determine whether the mean discrepancy significantly differed from zero. The VCI–PRI difference did not depart from zero (t(122) = −0.106, *p* = 0.916). Conversely, the WMI–PSI discrepancy was significantly negative (t(122) = −3.911, *p* = 0.002), confirming that, on average, WMI is lower than PSI by ≈6 points.

Importantly, the wide range and large standard deviation observed for both pairs of indexes indicate high intra-individual variability, suggesting that the apparent similarity (or difference) in group means does not fully reflect the marked heterogeneity of individual profiles.

Chi-squared tests of independence were conducted to examine whether clinically significant discrepancies (≥15 points) differed across diagnostic groups. For VCI–PRI, the association was not significant (χ^2^(2) = 4.10, *p* = 0.129), indicating no reliable group differences. Similarly, no significant association emerged for WMI–PSI discrepancies (χ^2^(2) = 1.21, *p* = 0.547). All expected cell frequencies exceeded 5, supporting the validity of the analyses. Overall, the likelihood of presenting a marked index discrepancy did not differ significantly among the AD, MD, and RD groups.

### 3.2. Latent Profile Analysis

**Figure 2:**
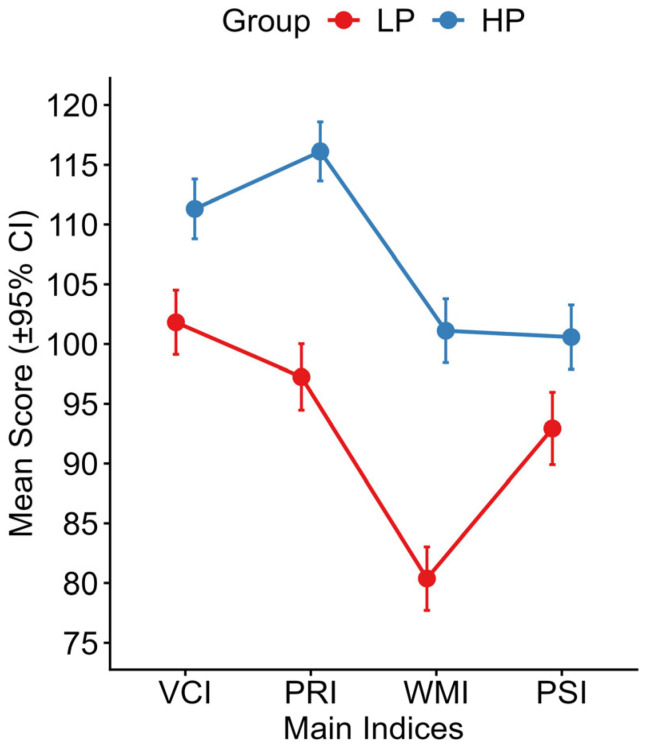
WAIS-IV indices scores for LP and HP groups. *Note*. LP = Low Profile group; HP = High Profile group; VCI = Verbal Comprehension Index; PRI = Perceptual Reasoning Index; WMI = Working Memory Index; PSI = Processing Speed Index. Error bars indicate 95% confidence intervals.

LPA fit indexes are presented in Table S1. The fit indexes produced a partially inconsistent pattern across the criteria: BIC, CAIC, and KIC supported a two-profile solution. AWE index favored the most parsimonious (one-profile) solution, and AIC, SABIC, and CLC favored the most complex (eight-profile) solution. These solutions were not considered optimal because they either failed to capture heterogeneity (one-class) or produced profiles with questionable interpretability and small classes (eight-class). We relied on the analytic hierarchy process (AHP), which integrates fit indexes [57], indicating that the two-profile solution (Figure 2) provided the best compromise between fit and parsimony and avoided small classes and therefore suggested better stability. This model showed a moderate classification accuracy (entropy = 0.65). The smallest profile included 49.6% of participants (*n* = 61), indicating a balanced distribution across profiles.

The two profiles’ indices summaries are detailed in Table 3. The first profile (*n* = 61; 49.6%), dubbed the “Low Profile” (LP), is characterized by adequate verbal (VCI) and visuo-perceptual abilities (PRI) but with significantly weaker working memory (WMI) and a processing speed (PSI) that, although within the normative range, does not fully compensate for the deficient area. Within-profile analysis for the “Low Profile” was explored using a one-way repeated-measures ANOVA, which revealed a highly significant effect of the cognitive domain on performance (F(3, 180) = 41.13, *p* < 0.001), with a large, generalized effect size (η^2^G = 0.344). Post hoc pairwise *t*-tests with Bonferroni’s correction highlighted a specific “drop” in working memory: WMI was significantly lower than all other indices: VCI (t(60) = 10.40, *p* < 0.001), PRI (t(60) = 8.71, *p* < 0.001), and PSI (t(60) = −6.21, *p* < 0.001). Additionally, VCI was also significantly higher than PSI (t(60) = 4.78, *p* < 0.001). No statistically significant differences were found between VCI and PRI (t(60) = 2.04, *p* = 0.277) or between PRI and PSI (t(60) = 2.09, *p* = 0.244).

The second profile (*n* = 62; 50.4%), dubbed the “High Profile” (HP), exhibits a general cognitive efficiency, with specific strengths in verbal (VCI) and perceptual reasoning (PRI) and with working memory (WMI) and processing speed (PSI) fully within the adequate range. Within-profile analysis for the “High Profile” was conducted using a non-parametric approach, as assumption testing revealed a significant violation of the normality assumption for PSI (Shapiro–Wilk *p* < 0.001). Friedman’s test indicated significant differences across the four domains (χ^2^(3) = 63.5, *p* < 0.001) with a moderate(Kendall’s *W* = 0.34). Wilcoxon’s pairwise signed-rank tests with Bonferroni’s correction revealed a different pattern of strengths and weaknesses: VCI and PRI represented the primary strengths of this profile, with PRI being significantly higher than VCI (V = 550, *p* = 0.044). Both VCI and PRI were significantly higher than the proficiency indices WMI and PSI: VCI differed significantly from WMI (V = 1633, *p* < 0.001) and PSI (V = 1624, *p* < 0.001). Similarly, PRI showed significant discrepancies when compared to WMI (V = 1788, *p* < 0.001) and PSI (V = 1724, *p* < 0.001). There was no significant difference between WMI and PSI (V = 937, *p* = 1).

Independent between-profile comparisons revealed that the HP significantly outperformed the LP across all indices: Student’s *t*-tests revealed a significant difference between profiles in VCI (t(121) = −5.05, *p* < 0.001), PRI (t(121) = −9.96, *p* < 0.001) and WMI (t(121) = −10.79, *p* < 0.001). Normality assumption was significantly violated for PSI in Profile 2 (Shapiro–Wilk *p* < 0.001); therefore, the non-parametric Mann–Whitney U-test was conducted, revealing a significant difference between profiles in PSI (W = 1249, *p* = 0.001). The largest effect sizes are observed in PRI (*d* = −1.80) and WMI (*d* = −1.95).

We next examined how diagnostic subgroups (i.e., AD, RD, and MD) were distributed across the emerged profiles using a chi-squared test. The results indicated a significant association between the profiles and the diagnostic group (χ^2^(2) = 19.70, *p* < 0.001). Specifically, the MD group was substantially overrepresented in the LP profile and underrepresented in the HP profile. Conversely, the RD group was markedly underrepresented in the LP profile and overrepresented in the HP profile. The AD group did not deviate from expected frequencies. The effect size of the chi-squared analysis was moderate (Cramer’s *V* = 0.40).

## 4. Discussion

Despite the high heterogeneity in functioning among adults with SLDs, even when they share the same diagnostic labels (e.g., dyslexia, dyscalculia, mixed disorders), research often treats this population as a unique group defined by core characteristics [10,16,42,59].

To overcome this limitation, it has recently been proposed that considering the specific cognitive functioning profile could be of great clinical utility in supporting individuals with SLDs, overcoming the limitations of nosographic categorization. Overall, the studies carried out so far show that the well-known discrepancy between good general reasoning skills and low efficiency that characterizes SLDs in school-age children also extends to the adult population [16]. Furthermore, unsurprisingly, in cases of mixed disorders (i.e., when reading, writing, and/or calculation disorders co-occur), the profile is similar to that shown by individuals with a single reading disorder, albeit with a greater performance gap [60]. The question we wanted to address in the present study was to understand how much the functioning profile may differentiate SLD subgroups, paying attention to the frequency of cognitive discrepancies within and between subgroups and, on these grounds, to reflect on the clinical validity of adopting the SLD categorical labelling with adult individuals.

### 4.1. General Cognitive Patterns in University Students with SLD

The present study examined cognitive variability among university students with SLD, analyzing both diagnostic subgroups and latent cognitive profiles. Across the entire sample, a consistent discrepancy emerged between reasoning-related abilities and cognitive efficiency indices, with the General Ability Index (GAI) significantly higher than the Cognitive Proficiency Index (CPI). This pattern is consistent with previous studies on adults with SLD, which have reported relatively preserved reasoning abilities alongside persistent weaknesses in working memory and processing speed [16,17].

Similarly, developmental research on children and adolescents with SLD reports cognitive weaknesses in Working Memory Index (WMI) and Processing Speed Index (PSI), even when reasoning abilities are average or above-average [18,32]. These findings support the idea that SLD-related cognitive differences are enduring rather than transient developmental delays [16]. Thus, adding to existing evidence, the present findings suggest that this cognitive profile is not restricted to childhood but persists in adulthood, even among individuals who have successfully attained higher education. It is worth noting that, in the face of persistent learning difficulties, the assessment of cognitive functioning is not only mandatory to exclude borderline or intellectual disability (as stated by the American Psychiatric Association’s Diagnostic and Statistical Manual of Mental Disorders 2013), but it is also compelling to accurately interpret low performance in reading, writing, and calculation tests. Therefore, on the one hand, good intellectual abilities are to be expected (otherwise, an SLD diagnosis should not be made), and on the other hand, SLD appears to involve systematic strengths in higher-order reasoning processes alongside persistent vulnerabilities in cognitive efficiency domains. However, one may suggest that the prevalence of strong reasoning abilities alongside low efficiency may be partially determined by the structure of the Wechsler scale. Indeed, the Cognitive Proficiency Index combines verbal working memory, which is often considered a weakness in learning disorders (e.g., [61,62]), with timed visuo-motor and visuo-perceptual tasks that are sensitive to visuo-attentional and executive slowness, both of which are frequently observed in learners who struggle [63,64]. Research has shown that low auditory working memory specifically contributes to academic achievement in university students with SLD, compared to ADHD or other clinical control groups [65]. Thus, given that the Wechsler WMI primarily assesses auditory working memory (i.e., the Backward Digit Span, Letter–Number Sequencing and Arithmetical Reasoning subtests) but also includes an assessment of auditory short-term memory (i.e., the Forward Digit Span subtest), the index of a marked fragility in this domain is not surprising. While the Wechsler scale provides a partial assessment of the working memory system, it confirms long-standing weaknesses by exploring the most informative components for SLD.

### 4.2. Differences Across Diagnostic Subgroupse

Although the general cognitive pattern was broadly consistent across groups, some meaningful differences emerged among diagnostic subtypes. Students with predominant reading disorder (RD) showed significantly higher overall cognitive scores compared to those with mixed disorder (MD), particularly in reasoning and memory indices. In contrast, the MD group displayed the lowest WMI scores and a pronounced discrepancy between working memory and processing speed.

These findings are consistent with developmental evidence indicating that mixed SLD profiles are typically associated with greater cognitive and functional severity than selective disorders [32]. The present results extend this observation to adulthood, suggesting that the co-occurrence of multiple learning impairments may reflect more pervasive underlying cognitive vulnerabilities.

In the arithmetic disorder (AD) group, a distinct pattern emerged in the relationship between verbal comprehension and perceptual reasoning, with Verbal Comprehension Index (VCI) scores exceeding Perceptual Reasoning Index (PRI) scores. Although this effect was observed in a relatively small subgroup, it is noteworthy given the limited literature on dyscalculia in adulthood [38]. Moreover, it is worth noting that this profile appears to characterize younger dyscalculics, as reported in large-scale analyses of individuals with SLD [32]. Here, PRI scores were relatively lower than VCI scores, though never below the norms, in the subgroup with arithmetical disorder, in line with findings indicating perceptual and visuospatial deficits in children with dyscalculia (e.g., [66,67]). In sum, these results may strengthen the hypothesis that domain-specific weaknesses in visuospatial or nonverbal reasoning processes are often implicated in mathematical learning difficulties.

Overall, subgroup differences were modest, and no sharply differentiated cognitive signatures emerged across diagnostic categories. This is consistent with the literature, which suggests that SLD diagnostic labels often capture overlapping and mixed cognitive patterns rather than distinct and isolated deficits [16,42].

### 4.3. Intra-Individual Variability and Cognitive Discrepancies

A key finding of the present study concerns the high intra-individual variability observed across cognitive domains, which challenges the traditional reliance on group-level averages in SLD research. Large discrepancies were observed both between reasoning indices (VCI–PRI) and between cognitive efficiency indices (WMI–PSI), with substantial proportions of participants showing clinically meaningful differences.

At the group level, the only systematic discrepancy was observed between WMI and PSI, with working memory scores significantly lower than processing speed scores. This pattern is consistent with previous research highlighting working memory as a core cognitive vulnerability in individuals with SLD, even in adulthood [16,17].

However, the wide range of individual discrepancy scores observed in our sample reached differences of up to 50 standard points between indices and underscores a substantial heterogeneity of cognitive profiles that aggregate data often obscure. For instance, the absence of a significant group-level bias in VCI–PRI discrepancies does not indicate a uniform level of reasoning abilities; rather, individuals appear to present with highly idiosyncratic configurations where verbal and nonverbal strengths often balance each other out in the total average.

This high intra-individual variability supports earlier claims that adult SLD cannot be adequately described by a unitary deficit model [42]. We argue that the modest numerical differences often reported in the literature between cognitive indices may, in some cases, be an artifact of averaging divergent individual profiles. When some students exhibit a marked “verbal-drift” and others a “perceptual-drift,” these patterns partially cancel each other out in aggregate analyses. By exploring these at a subject level, our findings emphasize the importance of looking beyond mean-based trends, as the “average” profile may not be representative of any SLD.

### 4.4. Latent Cognitive Profiles and Their Relation to Diagnostic Categories

Given the limited explanatory power of diagnostic categories for cognitive profiles, we aimed to identify unobserved subgroups using Latent Profile Analysis. This method identified two cognitive profiles: a lower-functioning profile characterized by relatively weak working memory and lower overall index scores and a higher-functioning profile marked by stronger reasoning abilities and more balanced cognitive efficiency with notable average working memory resources. Importantly, these profiles were distributed across diagnostic categories rather than aligning neatly with them.

Students with MD disorder were overrepresented in the lower cognitive profile, whereas those with predominant reading disorder were more likely to fall into the higher cognitive profile. This pattern is consistent with the subgroup analyses and, non-surprisingly, further supports the notion that mixed SLD conditions are associated with broader cognitive vulnerabilities [32].

Similarly, in the face of broader cognitive weakness, an individual is more likely to experience difficulties in multiple learning domains and, consequently, receive multiple SLD diagnoses (e.g., reading, spelling and calculation). MDs are harder to compensate for due to weaker cognitive resources [25,26], so we expect their learning profile to remain relatively consistent over time. Future longitudinal studies will provide valuable insight into the stability of learning profiles in selective and mixed disorders.

As previously reported, individuals with RD were overrepresented in the high-functioning profile, which is consistent with studies investigating the neuropsychological profile of university students with dyslexia. Despite recognition of internal variability, the literature on university students highlights that when an isolated reading deficit is present, it occurs alongside broader cognitive resources [68].

Interestingly, individuals with RD were more likely to exhibit a high cognitive profile, whereas individuals with AD were equally likely to exhibit either a high or a low cognitive profile. While the small size of this subgroup means that conclusions should be drawn with caution, we believe that this may indicate that clinical variability within the predominant arithmetic disorder group is even higher than in the other groups. This is due to the high heterogeneity of AD in terms of underlying functional impairments [68,69,70]. As widely recognized in the literature, AD may reflect either domain-specific deficits (i.e., quantitative information processing) and/or domain-general deficits (i.e., visuospatial and verbal abilities), leading to highly variable phenotypes [71,72]. Furthermore, an additional factor contributing to inter-individual variability within this group may be the heterogeneity in the clinical assessment and operationalization of mathematical disorder in adulthood.

In conclusion, the moderate classification accuracy and the partial overlap between diagnostic and cognitive groupings suggest that diagnostic labels alone do not fully capture the underlying cognitive heterogeneity. This finding echoes both the early and contemporary literature emphasizing the importance of identifying cognitive subtypes within SLD populations [29,42].

In this perspective, it is important to consider the recent transition from ICD-10 to ICD-11 (Developmental Learning Disorder, 6A03). While our data were originally collected under the ICD-10 framework, the findings strongly resonate with the dimensional shift promoted by ICD-11. By replacing isolated diagnostic codes with a unified category and specifiers for area of learning impairment, ICD-11 acknowledges the high rates of internal comorbidity and the persistent, lifelong nature of these disorders. In line with dimensional approaches to neurodevelopmental disorders, our identification of latent profiles that transcend traditional labels provides empirical support for this diagnostic paradigm and indicates that cognitive functioning in adults with SLD is better described as a continuum of profiles rather than as discrete, diagnosis-bound categories [1]. We ought to suggest that a more fluid, profile-based classification is better suited to capture the actual functioning of adults with SLD compared to the categorical boundaries of the past.

### 4.5. Implications for Adult SLD and Higher-Education Contexts

The present findings have several implications for understanding SLD in adulthood, particularly within university settings. First, the persistence of working memory weaknesses and the consistent GAI–CPI discrepancy confirm that core cognitive characteristics of SLD remain detectable even among academically successful individuals [16,17]. Moving beyond the diagnostic labels, it is this specific configuration of reasoning and efficiency that dictates the functional cost of academic tasks. These findings suggest that the impact of SLD in university is not merely a matter of impaired reading or calculating but of how cognitive resources are allocated during complex learning.

Second, the marked intra-individual variability observed across cognitive indices suggests that accommodations and support strategies should be tailored to individual cognitive profiles rather than based solely on diagnostic labels. This is especially relevant in higher-education contexts, where academic demands are heterogeneous and often require flexible cognitive resources. These results suggest that standardized accommodations that are meant for everyone indistinctly may be poorly targeted.

Finally, the identification of latent cognitive profiles highlights the potential value of profile-based approaches in both research and clinical practice. Such approaches may help to bridge the gap between diagnostic classifications and the functional cognitive characteristics that shape academic performance and adaptive outcomes [29]. University support systems should therefore shift from a label-based model to a profile-based model, recognizing that a single diagnosis can mask fundamentally different individuals.

## 5. Conclusions

Overall, the present study provides evidence of substantial cognitive heterogeneity among university students with SLD, even within the same diagnostic subgroup. This strengthens the conceptualization of SLD as a heterogeneous and dimensional condition, as emphasized by DSM-5 and the contemporary literature [1,16]. The fact that our sample consists exclusively of university students, a relatively high-functioning and selective subgroup of adults with SLD, limits the generalizability of the findings to the broader adult SLD population, which is likely to include individuals with more severe or less compensated profiles [29]. A second limitation is the retrospective nature of clinical documentation, which introduces high variability in assessment procedures and reporting practices. In fact, the methodology, procedures, and formal reporting of clinical services differ significantly, despite updated national guidelines to which any expert licensed psychologist is requested to refer to (e.g., [44]). Yet, secondary analysis of archival data, originally collected for clinical assessment, represents a common and informative methodology in research on the SLD population, providing access to a large corpus of data [16,32,73]. Therefore, whilst fully aware of the limits of this method, we believe that, when focusing on a specific population, such as university students from a single institution, document analysis can provide valuable insights. That said, prospective studies in more diverse adult samples with enriched cognitive and learning assessments remain a priority for future research.

Despite these limitations, the findings of the present study provide useful insights for both clinical and educational contexts. Across the entire sample, reasoning abilities were generally preserved, whereas working memory and, to a lesser extent, processing speed emerged as relative weaknesses. This pattern is consistent with the previous literature and supports the notion that core cognitive characteristics of SLD persist into adulthood. It is worth noticing that the LPA showed that for a proportion of university students with SLD (i.e., the high-functioning profile), working memory no longer constitutes a weak domain. More critically, differences across diagnostic subgroups were modest, although mixed SLD profiles were associated with lower cognitive efficiency and greater weaknesses in working memory. Importantly, Latent Profile Analysis revealed cognitive subgroups that only partially overlap with diagnostic categories, highlighting the limited explanatory power of diagnoses alone.

The findings suggest that traditional categorical labels for specific learning disorders (SLDs) have limited explanatory power, as they often obscure significant cognitive heterogeneity and variability within the same diagnostic group. Since diagnostic categories frequently capture overlapping cognitive patterns rather than distinct deficits, clinical assessment and support strategies should adopt a dimensional, profile-based approach.

In this regard, an up-to-date profile of a student’s functioning, covering both cognitive abilities and different areas of learning and providing insights into how these have developed over time, is far more useful than a diagnostic classification when it comes to determining which forms of support might be effective for that student. Based on our findings, for example, we know that the same label of “prevalent arithmetic disorder” can apply to individuals with very different cognitive abilities, ranging from outright cognitive inefficiency (i.e., low cognitive profile) to cognitive resources that fall entirely within the normative range (i.e., high cognitive profile). In the former, it is likely that the “extra-time” accommodation will not be sufficient to reduce cognitive costs or overcome efficiency constrains. Instead, to foster long-term independence, support should prioritize self-regulatory tools such as metacognitive coaching and memory externalization strategies [74]. In conclusion, only by moving beyond discrete labels and focusing on an individual’s unique combination of cognitive strengths and weaknesses can we develop truly effective, personalized support for adults with SLDs.

## Figures and Tables

**Table 1:** Demographic data and descriptive statistics of WAIS-IV and WISC-IV indices scores for the total sample and the RD, AD and MD groups.

	Total (*n* = 166)	RD (*n* = 79)	AD (*n* = 24)	MD (*n* = 63)	
	M ± SD (Range)	M ± SD (Range)	M ± SD (Range)	M ± SD (Range)	*p*-Value
**Age, years**	17.36 ± 2.04 (13–25)	17.7 ± 2.05 (13–24)	17.0 ± 2.22 (13–21)	17.2 ± 1.98 (13–25)	0.322
**Gender (M/F)**	59/107	43/36	1/23	15/48	<0.001
**GAI**	108.4 ± 11.97 (85–145)	112.01 ± 12.36 (85–145)	104.62 ± 10.66 (85–124)	105.32 ± 10.72 (85–133)	
**CPI**	92.08 ± 12.92 (57–132)	96.06 ± 11.93 (66–132)	92.5 ± 12 (66–115)	86.92 ± 12.82 (57–115)	
**VCI**	107.02 ± 12.2 (80–144)	108.58 ± 12.5 (86–144)	109.04 ± 13.15 (80–129)	104.3 ± 11.1 (82–129)	
**PRI**	108.04 ± 14.19 (77–139)	112.82 ± 13.38 (81–139)	99 ± 12.33 (79–121)	105.49 ± 13.67 (77–132)	
**WMI**	90.22 ± 14.37 (57–126)	95.78 ± 12.95 (66–126)	89.58 ± 15.11 (63–120)	83.49 ± 12.99 (57–117)	
**PSI**	96.42 ± 12.75 (65–139)	97.57 ± 13.21 (65–139)	98.38 ± 9.61 (75–114)	94.22 ± 13.06 (65–128)	

*Note*. RD = predominant reading disorder; AD = predominant arithmetic disorder; MD = mixed learning disorder; GAI = General Ability Index; CPI = Cognitive Proficiency Index; VCI = Verbal Comprehension Index; PRI = Perceptual Reasoning Index; WMI = Working Memory Index; PSI = Processing Speed Index; M = mean standard score; SD = standard deviation.

**Table 2:** Descriptive statistics of WAIS-IV indices scores for the subsample and RD, AD and MD groups.

	Subsample (*n* = 123)	RD (*n* = 57)	AD (*n* = 15)	MD (*n* = 51)
WAIS-IV Indices	M ± SD (Range)	M ± SD (Range)	M ± SD (Range)	M ± SD (Range)
**VCI**	106.6 ± 11.41 (82–141)	107.86 ± 11.42 (90–141)	108.73 ± 12.09 (90–129)	104.57 ± 11.09 (82–129)
**PRI**	106.76 ± 14.11 (77–139)	112.05 ± 12.97 (81–139)	95.27 ± 12.3 (79–121)	104.22 ± 13.29 (77–131)
**WMI**	90.83 ± 14.87 (57–126)	97.89 ± 13.13 (66–126)	87.13 ± 16.03 (63–120)	84.02 ± 12.88 (57–117)
**PSI**	96.79 ± 12.04 (67–139)	98.21 ± 12.59 (67–139)	98.6 ± 9.75 (75–114)	94.67 ± 11.9 (72–128)

*Note*. RD = predominant reading disorder; AD = predominant arithmetic disorder; MD = mixed learning disorder; VCI = Verbal Comprehension Index; PRI = Perceptual Reasoning Index; WMI = Working Memory Index; PSI = Processing Speed Index; M = mean standard score; SD = standard deviation.

**Table 3:** Descriptive and inferential statistics of WAIS-IV indices scores for the “High Profile” and “Low Profile” groups.

	LP (*n* = 61)	HP (*n* = 62)		
WAIS-IV Indices	M ± SD	M ± SD	*p*-Value	Effect Size
**VCI**	101.82 (10.73)	**111.31 (10.08)**	<0.001 ^a^	−0.91 ^b^
**PRI**	97.25 (11.07)	**116.11 (9.91)**	<0.001 ^a^	−1.80 ^b^
**WMI**	80.38 (10.56)	**101.11 (10.75)**	<0.001 ^a^	−1.95 ^b^
**PSI**	92.93 (12.07)	**100.58 (10.81)**	0.001 ^c^	0.29 ^d^
***p*-Value**	<0.001 ^e^	<0.001 ^g^		
**Effect size**	0.34 ^f^	0.34 ^h^		

*Note.* Values representing the highest scores are given in bold. Means and standard deviations for indices scores across profiles: VCI M = 106.6 (SD = 11.41), PRI M = 106.76 (SD = 14.11), WMI M = 90.83 (SD = 14.87), PSI M = 96.79 (SD = 12.04). LP = Low Profile group; HP = High Profile group; VCI = Verbal Comprehension Index; PRI = Perceptual Reasoning Index; WMI = Working Memory Index; PSI = Processing Speed Index; M = mean standard score; SD = standard deviation. ^a^ Calculated using Student’s *t*-test; ^b^ Cohen’s *d*; ^c^ calculated using the non-parametric Mann–Whitney U-test (Wilcoxon’s rank-sum); ^d^ rank-biserial correlation, ^e^ calculated using one-way repeated-measures ANOVA; ^f^ generalized eta-squared (η^2^G), ^g^ calculated using the non-parametric Friedman test; ^h^ Kendall’s *W*.

## Data Availability

The data used in this study consists of documentary sources and archival materials. Further information about the sources analyzed is available from the corresponding author upon reasonable request.

## Supplementary Materials

The following supporting information can be downloaded at: https://www.mdpi.com/article/10.3390/brainsci16040404/s1, Table S1: Latent Profile Analysis model fit summary.

## Abbreviations

The following abbreviations are used in this manuscript:

SLD	Specific Learning Disorder
DSM-5	Diagnostic and Statistical Manual of Mental Disorders, 5th Edition
ICD-11	International Classification of Diseases, 11th Revision
ICD-10	International Classification of Diseases, 10th Revision
WAIS-IV	Wechsler Adult Intelligence Scale, Fourth Edition
WISC-IV	Wechsler Intelligence Scale for Children, Fourth Edition
GAI	General Ability Index
CPI	Cognitive Proficiency Index
VCI	Verbal Comprehension Index
PRI	Perceptual Reasoning Index
WMI	Working Memory Index
PSI	Processing Speed Index
FSIQ	Full-Scale IQ
RD	Predominant Reading Disorder
AD	Predominant Arithmetic Disorder
MD	Mixed Learning Disorder
LMM	Linear Mixed-Effects Model
LPA	Latent Profile Analysis
