# Supplementary material for: What Lies Behind Diagnostic Labels? High Intra-Individual Variability Is the True Cognitive Signature of University Students with Specific Learning Disorders
Source: Brain Sci. 2026 Apr 10;16(4):404. doi: 10.3390/brainsci16040404 (PMC13115440; doi:10.3390/brainsci16040404)
Supplement: Supplementary file 1 [file brainsci-16-00404-s001.zip › brainsci-4216183-supplementary.pdf]

Table S1. Latent Profile Analysis model fit summary.

Model	Log likelihood	AIC	BIC	SABIC	AWE	CAIC	CLC	KIC	Entropy	Smallest profile N	Smallest profile %	Prob.Min.	Prob.Max.
**1**	-1959.07	3934.14	3956.64	3931.34	4017.13	3964.64	3920.14	3945.14	1	123	1	1	1
**2**	-1934.65	3895.31	3931.87	3890.76	4032.13	3944.87	3870.60	3911.31	0.65	61	0.5	0.9	0.9
**3**	-1931.5	3899.01	3949.62	3892.71	4089.08	3967.62	3864.17	3920.01	0.58	29	0.24	0.6	0.91
**4**	-1924.64	3895.28	3959.96	3887.23	4138.25	3982.96	3850.67	3921.28	0.69	16	0.13	0.73	0.9
**5**	-1921.75	3899.50	3978.24	3889.71	4195.60	4006.24	3844.89	3930.50	0.69	12	0.1	0.68	0.86
**6**	-1918.02	3902.04	3994.84	3890.50	4251.19	4027.84	3837.49	3938.04	0.73	9	0.07	0.65	0.94
**7**	-1907.4	3890.80	3997.66	3877.51	4292.95	4035.66	3816.38	3931.80	0.79	6	0.05	0.6	0.97
**8**	-1899.16	3884.32	4005.24	3869.28	4339.52	4048.24	3799.96	3930.32	0.82	3	0.02	0.64	0.97

Note. n = 123; LPA = latent profile analysis; AIC = Akaike's Information Criterion; BIC = Bayesian Information Criterion; SABIC = Sample-Adjusted BIC; AWE = Approximate Weight of Evidence; CAIC = Consistent Aikake Information Criterion; CLC = Classification Likelihood Criterion; KIC = Kullback Information Criterion. Prob.Min.and Prob.Max reflect classification certainty of cases (cases are assigned to classes they have a high probability of belonging to).
